# Micro-drive Array for Chronic *in vivo* Recording: Tetrode Assembly

**DOI:** 10.3791/1098

**Published:** 2009-04-22

**Authors:** David P. Nguyen, Stuart P. Layton, Gregory Hale, Stephen N. Gomperts, Thomas J. Davidson, Fabian Kloosterman, Matthew A. Wilson

**Affiliations:** Department of Brain and Cognitive Science, MIT - Massachusetts Institute of Technology; Picower Institute for Learning and Memory, MIT - Massachusetts Institute of Technology

## Abstract

The tetrode, a bundle of four electrodes, has proven to be a valuable tool for the simultaneous recording of multiple neurons in-vivo.  The differential amplitude of action potential signatures over the channels of a tetrode allows for the isolation of single-unit activity from multi-unit signals.  The ability to precisely control the stereotaxic location and depth of the tetrode is critical for studying coordinated neural activity across brain regions.  In combination with a micro-drive array, it is possible to achieve precise placement and stable control of many tetrodes over the course of days to weeks.  In this protocol, we demonstrate how to fabricate and condition tetrodes using basic tools and materials, install the tetrodes into a multi-drive tetrode array for chronic in-vivo recording in the rat, make ground wire connections to the micro-drive array, and attach a protective cone onto the micro-drive array in order to protect the tetrodes from physical contact with the environment.

**Figure:**
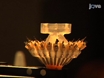


## Protocol

### 1. Fabrication of tetrodes

1. Begin the construction of one tetrode by obtaining a 50 cm long, polyimide insulated, ni-chrome wire with a 12.5 micron diameter core.  Fold the wire in half.  Run your fingers along the pair to make them stick together.  Make sure the pair of wires have good contact throughout their entire length.
2. Fold the pair again by holding the two ends together.  Make sure that the loop formed on one end is not kinked. Cut the four wires on the non-looped end so that the four tips are aligned.
3. For the next step, you will need a modified alligator clip, a motorized turning device, and a horizontal bar above the turning device.  The alligator clip is modified by gluing a plastic bar to the base of the clip.  Where the wire tips are exposed, clamp the four wires together with the modified alligator clip.  Hang the loop of wires over the horizontal bar. Place the alligator clip into the motorized stage.
4. Apply 80 clock-wise twists followed by 40 counter clock-wise twists to the wire bundle over the course of approximately three minutes.  These parameters can be varied according to your needs.
5. After tetrode twisting is completed, fuse the wires together by heating from three different angles with a heat gun (420° C or 790° F), using medium-low flow.  For each angle, begin 1-2 cm below where the wire bundle splits, run the heat gun down and up at a 2 cm distance from the wires for about 5 seconds.  Use caution as high temperatures will completely melt the insulation and lead to short circuits.
6. Now that the wires have fused together, remove the tetrode from the twisting apparatus by gently lifting the alligator clip to relieve tension on the tetrode, and cut the tetrode near the alligator clip.  At the other end, cut the loop such that there are four non-bonded strands of wire of equal length.
7. Next, separate the individual strands at the top by gently bending the wires with a soft tipped tweezer.
8. The tetrode is now ready for loading into the micro-drive array.  Make 21 to 25 more tetrodes and store them in a dust free box until it is time for the tetrode loading process.

### 2. Loading the tetrodes and ground wires into the micro-drive array

1. To proceed, you will need a complete micro-drive array.  If you have not yet built one, refer to the video 'Micro-drive array for chronic in-vivo recording: drive fabrication.'  In our design plan, the tetrode will be attached at one end to connector hardware, and will run through the polyimide carrier tube in the micro-drive such that the electrode tips extend below the base of the micro-drive array.
2. Before beginning, build a drive holder that can be attached to the connector board on one end, and on the other end, can be clamped by a panavise.  In this example, the drive holder is a mill-max connector glued to one end of an X-ACTO knife handle (Figure 1c).
3. Holding the tetrode with soft tipped tweezers and using a stereoscope, push the tip of the tetrode into the polyimide carrier tube of one of the micro-drives.  Push the tetrode into the tube until the individual electrode wires are close to the connector board at the top of the drive array.  Be careful not to kink or bend the wires, as that will cause the tetrode to enter the brain at an angle, rather than perpendicular, and will weaken the integrity of the tetrode.
4. Gently feed the other end of the four wires into their respective holes in the electrode interface board using soft tipped tweezers. Again, avoid kinking or bending the wires.  With all four wires in place, push the gold pins into their holes with a pair of pliers that have a shortened lower jaw. When the pins are pushed into the hole, they will strip the wire insulation and create an electrical connection.
5. For later reference, keep track of the mapping between micro-drives and pin position on the electrode interface board.
6. Continue loading a total of 18 tetrodes.  The three remaining micro-drives on the array will be used to house the reference electrodes. Each reference electrode is made of a tetrode that uses only one of the four unbonded wire ends. To attach the reference electrodes, follow the loading procedure but attach only one of the four electrodes to the respective reference electrode pin on the connector board.
7. If you are planning multi-site recordings, it may be worthwhile to plan out the connections from the tetrodes to the connector board ahead of time.  This should minimize the possibility of adjusting the wrong tetrode during experiments.
8. Once tetrode loading is completed, make two ground wires, one for the animal and one for the protective cone.  Cut one 6' long insulated steel wire (text: 0.005' diameter) and remove 3 mm of insulation, using metal tweezers, from each end of the wire.  Then cut a 4' long insulated steel wire (text: 0.005' diameter).  Remove 3 mm of insulation at one end and 1 cm at the other end.
9. Route the 6' ground wire through the hole on the side of the micro-drive array and up towards the interface board.  Connect the exposed wire to the electrode interface board with a gold pin at the designated ground hole.  This wire will connect to the animal's skull.
10. Route the 4' ground wire parallel to the previously installed ground wire.  Connect the 3 mm stripped end to the electrode interface board using a gold pin at another designated ground hole. This wire will later be connected to a protective cone, which will act as a small Faraday cage to reduce noise pickup. We are now finished connecting wires and can proceed with refining our tetrodes.

### 3. Setting the length of the tetrodes

1. Our goal in this process is to cut all the tetrodes so that they are slightly longer than the depth of the target brain structure.  In our example, the micro-drives are designed for a maximum travel of 5-6 mm, sufficient to reach many neocortical areas and the dorsal hippocampus of an adult rat. First, lower all the micro-drives so that the tetrodes are maximally exposed. Simultaneously cut all the tetrodes to lengths that are approximately 5 mm longer than the desired final length using a pair of sharp fine scissors.
2. For the final cut, prepare a pair of serrated scissors in a panavise.  Clamp one handle with a slight angle downwards and with serrations facing upwards, while leaving the other handle dangling.
3. Now completely withdraw all the tetrodes into their guide cannula by turning the micro-drives up.
4. Using a stereoscope, fully extend one tetrode out from its cannula.  Using a ruler, position the serrated scissors at the desired distance from the guide cannula.  Slowly and gently cut the tetrode with one smooth movement. After this tetrode has been cut, withdraw the micro-drive up by 3-4mm.  Repeat this process for each tetrode until all have been cut.  At this point you are ready for gold plating.

### 4. Gold plating of tetrodes and quality check

Gold plating the nichrome tetrode wires is critical for long term stable recordings.  It prevents corrosion and improves biocompatibility.

1. Setup the impedance meter in external mode and the current generator in DC check mode with audio output (Figure 1d).  Touch the two leads of the current generator to the six possible pairs of channels within each tetrode, and use the audio feedback to determine if shorts exist between pairs.  If a short exists, re-cut the tetrode with serrated scissors and test again.  If the problem persists, discard the tetrode and replace it.
2. When no shorts exist within all the tetrodes, move all the micro-drives down, extending all the tetrodes as far out as possible.
3. Modify the equipment configuration for checking electrode impedance (Figure 1e).  Now dip the tips of all the tetrodes into a bath of standard gold-plating solution.  The gold bath is electrically connected to the positive lead of the impedance meter.  Check the impedance of the electrode by touching the negative lead of the impedance meter to the corresponding pin on the connector board.
4. Measure and record the impedance of every wire on the micro-drive array.  Normal values range from 1-3 MOhm.
5. On the current generator, set the current between 1-3 μA.  Next, securely attach the negative lead of the impedance meter to the pin on the connector board.  Place the equipment into the electroplating configuration (Figure 1f).  On the impedance meter, quickly switch from normal mode, to bypass mode, and back.  While in bypass mode, current will pass through the tetrode and gold solution, and gold will be plated onto the electrode tip.  The impedance after each round of plating should be less than before. Repeat the current pulse if the impedance did not drop below 1 MOhm.  A conservative range of acceptable impedances are 250kOhm to 350 kOhm.  If the impedance drops below 200 kOhm, this may indicate a short circuit.
6. If after repeated pulses the impedance does not drop sufficiently, the electrode tip may be obstructed.  Re-cut the tetrode, check for shorts, and repeat the gold-plating process.  If re-cutting doesn't help, replace the tetrode and start again.
7. After plating all tetrodes, dip the tetrode tips briefly in ethanol, and let them dry for 3 to 5 minutes.
8. Test for short-circuits within tetrodes using the current generator with the tetrode tips exposed to air.  If a DC connection exists between two or more electrodes of a tetrode, re-cut the tetrode, and plate with gold again.
9. After gold-plating of all the tetrodes is complete, add a drop of medium thickness cyano-acrylate glue to the interface between each tetrode and its polyimide tubing.  This will secure the tetrode to its micro-drive and maintain the length of the tetrode.
10. As a final step, move all the tetrodes into the drive by turning all the screws up.

### 5. Attaching the protective cone

The protective cone's purpose is to shield micro-drives and the exposed tetrode wire from the environment.  It also provides support for handling during an experiment and reduces electrical noise.  We use a 3-D printed plastic cone (Reference Part I: drive fabrication).  However, it is possible to make the cone from other materials, like a bent piece of soda can.

1. Take the plastic cone and fix some aluminum foil tape to the inner or outer surface.
2. Next, drill a small hole (1.5 mm in diameter) through the cone and loosely attach a screw with a washer and nut inside the cone.  This will serve as an electrical passage for the ground wire.  We recommend using an 0-80 size screw and washer combination.
3. First, insert and partially turn three 1-72 size screws (3/16 inches in length) into the sides of the cone. With the drive array suspended, insert the cone over the drive.  Make sure both ground wires extend from the bottom of the drive array.  Wrap the exposed part of the 4' ground wire around one of the 1-72 screws several times and fasten the screw tightly.  Secure the cone to the drive base by tightening the remaining two cone screws.
4. Lastly, take the 1 cm stripped end of the short ground wire and wind it around the screw, in between the foil tape and washer.  Secure the ground by tightening the screw.  Now we have a micro-drive array that is at last ready for surgical implantation.

### 6. Representative results

1. Micro-drive array production is now complete.  Your micro-drive array is loaded with 18 tetrodes and 3 reference channels.  It is encased in a protective cone and cap for durability.  The finished micro-drive array with protective cone weighs 20g to 25g on average.
2. The finished drive array is implanted using standard surgical procedure.  As you can see, the drive is attached to a preamplifier chip and a bundle of wires that carry the signal to data acquisition hardware and software.

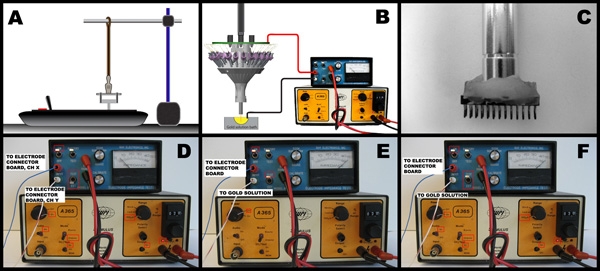
 **Figure 1:** **A)** Schematic of tetrode twisting configuration.  A horizontal bar suspends the unbonded electrode wires of the tetrode above the automated tetrode twisting device.  **B)**  Setup for tetrode impedance measurement and electrode plating.  When checking for short circuits, the circuit is different: the black wire is attached to another electrode channel on the connector board.  **C)** A close-up of a custom made micro-drive array holder tool.  The cylindrical bar is an X-Acto knife handle.  The connector is a 26-pin, double row, Mill-max connector.  The two pieces are bonded together using dental acrylic.  **D)** Configuration for short checking. Channel X and Channel Y are any two different channels of the four tetrode channels. **E)** Configuration for measuring electrode impedance.  The current source is 'off'.  The impedance meter is set to 'on' and 'test'.  **F)** Configuration for gold plating of electrode tips.  The current source is set to 'on', audio is 'on', mode is 'unipolar', DC Test is 'on', current is set to 2 microamps.

## Discussion

This protocol is the second installment of a two part protocol: “Micro-drive array for chronic in-vivo recording.”  The combination of the micro-drive array and tetrode is a powerful tool for obtaining simultaneous recordings of many neurons in an awake, behaving animal preparation.  Hopefully, we have provided you with all the necessary information for you will need to begin or improve electrophysiological recordings in your own laboratory.

The motorized tetrode twister device was custom designed and fabricated.  We have provided a vendor and part number that will allow you to order a device with similar functionality.  Alternatively, a motorized turning device may be constructed in-house simply by combining a motorized rotating bar magnet with a magnetized stirring rod that is attached to an alligator clip; this configuration would most likely require manual counting of rotations, manual reversal of rotation direction, and manual starting/stopping.

Some electrode interface boards do not have pin-and-hole mechanisms for connecting the electrode wire.  Instead, you may have obtained an interface board that has metal pins that extend out from the connector board; such metal pins are similar to those often found on standard electronic components.  We briefly outline a methodology for connecting the tetrode channels to metal pins.  First, remove the insulation on the tips of the four electrode wires of each tetrode.  This is accomplished by applying a flame to the tip of each electrode as briefly as possible using a handheld butane torch.  Obtain shrink wrap that will fit easily over the metal pin, and will squeeze the pin tightly after the shrink wrap has been heated.  Cut the shrink wrap tubing to the length of the metal pin.  Use capillary action to fill the shrink wrap with silver paint.  Insert the exposed tip of one electrode wire into the shrink wrap.  Gently slide the shrink wrap over the metal pin such that the electrode wire is between the pin and the wall of the shrink wrap.  Repeat these steps for the remaining three electrode channels of the tetrode.  Finally, with a side-to-side motion of a heat gun, heat the four pins covered in shrink wrap (with the electrode wires inside the shrink wrap).  At this point, you should have stable and reliable connections between the tetrode and connector board.

Throughout these fabrication procedures handle the electrode wires with care as very little force is required to damage them.  There are many steps in the tetrode and micro-drive array procedure and each step is as crucial as the others for obtaining high quality data.  The protocol described here has worked well for our laboratory considering the equipment available.  The resources in your own laboratory may be different, therefore, consider each step carefully and do not hesitate to change the protocol to meet your own requirements and constraints in order to achieve optimal performance.
